# The Evolution of British Orthopædics
*An address delivered at the Annual Meeting of the Winford Orthopædic Hospital, on 6th June, 1935.


**Published:** 1935

**Authors:** Humphry Rolleston

**Affiliations:** Physician Extraordinary to H.M. the King, Emeritus Regius Professor of Physic, Cambridge University


### The Bristol Medico-Chirurgical Journal

"*Scire est nescire, nisi id me*
*Scire alius sciret.*"

AUTUMN, 1935.

### THE EVOLUTION OF BRITISH ORTHOPÆDICS.*

BY

Sir Humphry Rolleston, Bart., G.C.V.O., K.C.B., M.D.,

*Physician Extraordinary to H.M. the King,*
*Emeritus Regius Professor of Physic, Cambridge University.*

The words orthopædy, orthopædics, and the like (ὀρθός = right, straight; παιδεία = rearing of children) date from 1741, when Nicolas Andry[1] (1658–1742) published *L'orthopédie, ou l'art de prévenir et de corriger dans les enfants les difformités du corps,* thus prophetically emphasizing the modern ideal of medicine, namely the prevention of disease as a higher aim than its cure. Andry was the dean, and a quarrelsome one, of the Paris Faculty of Medicine, and like his colleagues did not escape the frank caricaturists of

* An address delivered at the Annual Meeting of the Winford Orthopædic Hospital, on 6th June, 1935.

N

the period ; in *La politique du médecin de Machiavel, ou le chemin de la fortune ouvert aux médecins* (1746), by J. O. de La Mettrie (1709–51), a disgruntled Paris physician, Andry appeared as "Verminosus," an allusion justified by the nature of his writings and profitable practice, and not overtly applied to his personal state. This medical satire recalls Sir Samuel Garth's earlier mock Homeric poem, *The Dispensary* (1699), which gave an account of the dispute at the Royal College of Physicians of London, the prominent protagonists being presented under somewhat thinly-disguised pseudonyms. This Nicolas Andry must not be confused with his junior contemporary Nicolas Andry (1704– ? ), a charlatan and voluble advertiser of his anti-venereal urethral bougies. The first reference to the use of the word orthopædy in the *Oxford English Dictionary* is from the prospectus of the Royal Orthopædic Hospital, London, in 1840.

Like other future special branches of medicine the embryonic elements or genes of orthopædics were present in the corpus Hippocraticum, for example, the *scannum* (bench) for the reduction of dislocations. If the primitive splints applied to fractures can be justifiably dignified with the title of rudimentary orthopædic appliances, the history of the subject goes back nearly five thousand years more—to 5000 B.C. in Egypt (Stuck),[6] and, in fact, must have been one of the earliest forms of the surgical art (Young).[9] In his comprehensive review of " orthopædics before Stromeyer " Muirhead Little[2] indicated in detail the outstanding points of historical interest, such as the recommendation of tenotomy in the treatment of ankylosed and contracted joints made by Antyllus (*circa* A.D. 300) ; the work of Ambroise Paré (1510–90), the " father of modern surgery " ; of Francis Glisson

(1597–1677), whose classical *Tractatus de Rachitide seu Morbo puerili* (1650) naturally entailed a study of deformities; of William Cheseldon (1688–1752), who divided the sterno-mastoid muscle for torticollis, an operation which Dupuytren (1777–1835), the almost exact contemporary of Jacques Mathieu Delpech (1777–1832), "the scientific founder of orthopædics" (*vide* p. 146), performed by subcutaneous tenotomy in 1821. Jean André Venel (1740–91) of Geneva described by Garrison[1] as "the real originator of surgical orthopædics," was the first to start, at Orbe in the Canton Vaud, an orthopædic institute, an example copied in other parts of Switzerland and in Germany before this country followed suit in 1838 (*vide* p. 147). In these institutes patients stayed for considerable periods so as to obtain remedial treatment, especially by instruments. Bone-grafting dates back to 1682 when Meekren[3] (1611–82), a Dutch surgeon, successfully implanted a piece of a dog's skull into a soldier's cranium. The practical question of the formation of bone from periosteum later engaged the attention of James Syme (1799–1870) and John Goodsir (1814–67) of Edinburgh, and of Sir William Macewen (1848–1924) of Glasgow. Robert Chessher (1750–1831) of Hinckley, Leicestershire, was mechanically minded, and to some extent anticipated Zander; like Sir Robert Jones (1858–1933) he was most generous to his poor patients, and of him it is recorded that he died well-to-do, and still more rich in the good opinion of all who knew him.

A pioneer in operative orthopædics who indirectly awakened professional interest in this country in the subject was Georg Friedrich Louis Stromeyer (1804–76), "the father of modern military surgery in Germany," a poet and one who took the precaution of writing his own biography ; he performed his first subcutaneous tenotomy in 1831, fifteen years after this method of tenotomy had been originated by Delpech, professor of surgery at Montpellier and author of *L'orthomorphie* (1828), the earliest complete treatise on deformities of bones and joints. Delpech thus made a distinct advance on the open division of the tendo Achillis for club-foot, which had been performed in 1782 by Lorenz of Frankfort and in 1803 by Antonio Scarpa (1747–1832), professor of surgery at Pavia. In the year of Scarpa's death Delpech was murdered by a patient who believed that an operation for varicocele had rendered him impotent. Stromeyer popularized subcutaneous tenotomy as the remedy for all deformities due to muscular disorder ; his operation for equino-varus on William John Little (1810–94), afterwards physician to the London Hospital (1845–63), was the means of improving the position, and influencing the profession in this country to take an active interest in the practice, of orthopædics.

Little apparently had acute poliomyelitis when two years old, with resulting talipes equino-varus, and is said to have "entered on the physic line," to use an old phrase, in order to find a cure for the deformity which he shared with Sir Walter Scott and Lord Byron. At that time club-foot, being mainly in the hands of lay practitioners with a mechanical bent, was considered to be outside the scope of orthodox surgery. This position persisted, as is shown incidentally by Sir James Paget's[5] paper in 1867 on "Cases that bone-setters cure" which "more by luck than by wit are not a few." After education at Dover and St. Omer Little entered the Medical School of the London Hospital in 1828 and qualified three years later. He visited Montpellier to consult

Delpech, who discouraged the idea of operation. Probably about this time he unsuccessfully contested a vacancy for an assistant surgeon at the London Hospital; this failure may have deflected his professional ambition to internal medicine. When the great cholera epidemic of 1832 spread from Sunderland widely into this country Little had accompanied Frederick Cobb (1796–1883), then assistant physician to the London Hospital, to Newcastle-on-Tyne in order to study the disease which for the first time had established itself in England. In 1834 he went to Berlin, worked at physiology under Johannes Müller, and visited Stromeyer's Clinic at Hanover. Stromeyer, who in 1834 had modified Delpech's technique of tenotomy, divided Little's left tendo Achillis in July, 1836, with such a good result that the patient, full of enthusiasm, took the doctorate at Berlin in the following year with a dissertation "Symbolæ ad talipedem varum cognoscendum," the first monograph ever published on tenotomy. On returning to London in 1838 Little, sometimes called "the apostle of tenotomy," was assisted by R. W. Tamplin (1814–74), whose sister he had married, in establishing the "Orthopædic Institute," the first of its kind in this country, which later became the Royal National Orthopædic Hospital. In 1839 he brought out *A Treatise on the Nature of Club-foot and Analogous Distortions.* A long experience eventually modified his high opinion of the value of tenotomy, and he realized that indiscriminately employed it was a dangerous procedure. His third and fourth sons, Louis Stromeyer (1840–1911) and Ernest Muirhead, were both on the surgical staff of the Royal National Orthopædic Hospital, and the latter is the historian of orthopædic surgery. Congenital spastic paraplegia, to which Jakob Heine (1800–79) in 1840 had directed attention, was described by Little in his book *On the Nature and Treatment of Deformities of the Human Frame* (1853), and has been called Little's disease.

In America Philip Syng Physick (1768-1837) of Philadelphia, a favourite pupil of John Hunter, and the first American to hold a post in a London hospital (House Surgeon at St. George's Hospital), has been called "the father of American surgery." He was a pioneer in America of the treatment of bone tuberculosis by rest. But, as Nixon[4] has pointed out, the first surgeon in the country to practise and in 1813 to advocate the treatment of spinal caries by rest and sunlight was the little known Thomas Baynton (1761-1820), of Bristol. Another well-known American surgeon was Lewis Albert Sayre (1820-1900), known as "the father of American orthopædic surgery"; the plaster of Paris jackets he employed were very familiar fifty years ago in this country.

The modern science of orthopædics owes an enormous debt to the work of Hugh Owen Thomas and Sir Robert Jones. Hugh Owen Thomas (1834–91)[8] was the eldest of the seven children and the five sons of Evan Thomas (1804–84), a bone-setter in Liverpool, and came of a long ancestral line of Anglesey bone-setters of both sexes. Hugh Owen was apprenticed for four years to a medical man, studied at Edinburgh and qualified M.R.C.S. in 1857. Though he assisted his father for a time, he became independent, publicly contradicted the common belief that bone-setters possessed some occult art, and stated that "many of them are only successful competitors with shrines, relics and other nervine remedies." As he was a descendant of bone-setters and did not put his qualification after his name in his published works, it is perhaps not altogether surprising that he was widely regarded as a bone-setter, and was not fully recognized as a great pioneer until thirty years after his death. He was, like John Hilton (1804–78), the author of *Rest and Pain* (1863), an apostle of the value of rest as a healing factor, and was the inventor of splints which have made his name a hospital word all over the globe, and have suffered only from supposed improvements. In an appreciation written in 1916 Lynn Thomas[7] remarked, "On three occasions I have had the good fortune to find out in time that modifications I had approved were a degradation of the original." Thomas's nephew, partner and successor, Sir Robert Jones, worthily followed his lead, established the true value of his methods, and has been well described as "the guiding spirit of the British and American orthopædic services during the war" (Garrison).

The Winford Orthopædic Hospital must be congratulated on opening its doors, even wider than before, to children crippled, permanently or temporarily, by rheumatic inflammation of the heart. It is indeed most appropriate that this step should have been taken in Bristol, for the late Dr. Carey Coombs would have rejoiced to see its recent expansion; he had worked in a most devoted and unselfish manner for the well-being of the large number of children whose lives are spoiled and prematurely brought to a close by the insidious disease of the heart due to the rheumatic poison. It is satisfactory that his friend and senior colleague, Dr. F. J. Poynton, is now consulting physician to the hospital.

### REFERENCES.

Osgood, R. B., *The Evolution of Orthopædic Surgery*, St. Louis, 1925.

[1] Garrison, F. H., *Introduction to the History of Medicine*, pp. 342, 727, Phila., 1929.

[2] Little, E. M., in the *Robert Jones Birthday Book,* London, 1928.

[3] Meekren, J., quoted by Stuck.

[4] Nixon, J. A., *Proc. Roy. Soc. Med.*, Lond., 1915, viii. (Hist. Med. Sect.), p. 95.

[5] Paget, J., *Brit. Med. Jour.*, 1867, i. 1.

[6] Stuck, W. G., " Historic Backgrounds of Orthopedic Surgery," *Ann. Med. History,* New York, 1935, N.S. VII. 36–48.

[7] Thomas, J. L., " A Reconsideration of the Principles and Methods of Hugh Owen Thomas." *Brit. Med. Jour.*, 1916, ii. 71.

[8] Watson, F., *Hugh Owen Thomas: A Personal Study,* Oxford University Press, 1934.

[9] Young, J. K. A., *Manual and Atlas of Orthopædic Surgery,* London, 1906.

